# PIWI-RNAs Small Noncoding RNAs with Smart Functions: Potential Theranostic Applications in Cancer

**DOI:** 10.3390/cancers15153912

**Published:** 2023-08-01

**Authors:** Simona Taverna, Anna Masucci, Giuseppe Cammarata

**Affiliations:** 1 Institute of Translational Pharmacology (IFT), National Research Council (CNR), 90146 Palermo, Italy; 2 Department of Biomedicine, Neurosciences and Advanced Diagnostics, Institute of Clinical Biochemistry, Clinical Molecular Medicine, Laboratory Medicine, University of Palermo, 90127 Palermo, Italy; anna.masucci@community.unipa.it

**Keywords:** piRNAs, PIWI proteins, ncRNAs, extracellular vesicles, biomarkers, epidrugs

## Abstract

**Simple Summary:**

P-element-induced wimpy testis-interacting RNAs (piRNAs) are a novel class of small regulatory RNAs that often bind to PIWI proteins. First identified in animal germ line cells, piRNAs have key roles in germ line development. New insights into the functions of PIWI-piRNA complexes demonstrate that they regulate protein-coding genes. Aberrant piRNA expression has been also associated with different diseases, including cancer. Recently, piRNAs have been described in extracellular vesicles. EVs are one of the components of liquid biopsy, a revolutionary technique for detecting specific molecular biomarkers. This review focuses on piRNAs as potential biomarkers in different cancer types. Furthermore, piRNAs contained in extracellular vesicles could represent a new route for early diagnosis and therapies in a personalized medicine approach.

**Abstract:**

P-element-induced wimpy testis (PIWI)-interacting RNAs (piRNAs) are a new class of small noncoding RNAs (ncRNAs) that bind components of the PIWI protein family. piRNAs are specifically expressed in different human tissues and regulate important signaling pathways. Aberrant expressions of piRNAs and PIWI proteins have been associated with tumorigenesis and cancer progression. Recent studies reported that piRNAs are contained in extracellular vesicles (EVs), nanosized lipid particles, with key roles in cell–cell communication. EVs contain several bioactive molecules, such as proteins, lipids, and nucleic acids, including emerging ncRNAs. EVs are one of the components of liquid biopsy (LB) a non-invasive method for detecting specific molecular biomarkers in liquid samples. LB could become a crucial tool for cancer diagnosis with piRNAs as biomarkers in a precision oncology approach. This review summarizes the current findings on the roles of piRNAs in different cancer types, focusing on potential theranostic applications of piRNAs contained in EVs (EV-piRNAs). Their roles as non-invasive diagnostic and prognostic biomarkers and as new therapeutic options have been also discussed.

## 1. Introduction

Only 1–2% of transcriptomes are protein encoding. The latest evidence proved that a large scale of mammal genomes is transcribed to noncoding RNAs (ncRNAs). ncRNAs have emerged as an important class of genetic regulators, and their value in human diseases is becoming progressively more evident [1]. These molecules are often dysregulated in human cancers and can affect cancer progression via different mechanisms such as transcriptional and post-transcriptional modifications, epigenetics, and signal transduction [2]. The pivotal role of many ncRNAs in cancer is widely demonstrated, and they can be functionally classified into oncogenes or tumor suppressors [3,4,5]. ncRNAs are a heterogeneous family characterized by different lengths, biogenesis, and biological function, including (i) short ncRNAs as microRNAs (miRNAs), piwi-interacting RNAs (piRNAs), small nuclear-RNAs (snRNAs), and small nucleolar-RNAs (snoRNAs), and (ii) long ncRNAs, including circular RNAs (circRNAs) and long noncoding RNAs (lncRNAs) [4,6,7]. Among the short ncRNAs, piRNAs have emerged as the newest members of this family that are being recognized as important mediators of cell biology [8]. Several studies report that extracellular vesicles (EVs) deliver several biologically active molecules with a key role in cell–cell communication. EVs carry a wide range of cargo, such as ncRNAs, including piRNAs, which are selectively loaded into the vesicles [9]. Thus, strategies to specifically target ncRNAs contained in EVs (EV-ncRNAs) are an attractive therapeutic option [10]. This review focuses on piRNA functions in cancers and their potential clinical implications. Moreover, we discuss the potential of EV-ncRNAs as non-invasive diagnostic and prognostic biomarkers and as new therapeutic options.

## 2. Biogenesis of piRNAs

piRNAs were first found in germline cells and are considered critical regulators of germline maintenance. These animal-specific short-chain RNAs have a size of 24–32 nucleotides and a 2′-O-methylation at the 3′ end, a distinctive and exclusive feature of all piRNAs, and are associated with PIWI proteins. These proteins belong to the Argonaute protein family [9], which were discovered, for the first time, in *Drosophila melanogaster* ovarian germ cells and follicular cells [10]. piRNAs can bind DNA sequences of specific genes via complementary base pairing to silence transposons and regulate gene expression. The human genome contains over 30,000 piRNA genes that are mainly derived from intergenic regions. According to multiple origins, piRNAs are divided into three subclasses: mRNA-derived, lncRNA-derived, and transposon-derived piRNAs [11]. piRNAs transcribed from transposons are known as “piRNA clusters”. These clusters are mainly located in the pericentromeric and sub-telomeric parts of the chromosomes. piRNA clusters are transcribed to form piRNA precursors via bidirectional or unidirectional transcription. Moreover, piRNAs can be generated from mRNA 3′ untranslated region (3′ UTR) and some long non-coding regions in the genome [12]. The mechanism of piRNA production includes two steps: a primary and a secondary amplification cycle described as the “ping-pong cycle”, in which piRNAs are bound to PIWI proteins [13]. In primary amplification, newly transcribed piRNAs are exported through the nuclear envelope, processed, and matured. In the cytoplasm, the secondary structures are resolved via RNA helicase Armitage (Armi). After, piRNA precursors are cleaved by mitochondria-associated endonuclease Zucchini (Zuc) and transformed into pre-piRNAs with a 5′ monophosphate. Then, pre-piRNAs are loaded onto PIWI proteins and cut at the 3′ ends by a 3′ to 5′ exonuclease, Nibbler (Nbr) [12]. After, 3′ terminal ends are methylated at 2′ oxygen by RNA2′-O-methyltransferase Hen1 [13]. piRNAs produced in this way are named primary piRNAs. In secondary amplification, piRNAs’ generation is increased with the involvement of Argonauta 3 (Ago 3) and Aubergine (Aub) proteins. Aub binds to antisense strand piRNAs and cleaves sense piRNA precursors, giving rise to sense piRNAs bound by Ago3. In contrast, Ago3 binds to sense-strand piRNAs and cleaves antisense piRNA precursors, producing antisense piRNAs that load onto Aub [14]. The round of cleavage repeats and produces several piRNA molecules. These piRNAs are then bound by PIWI proteins and transported back to the nucleus to silence target genes. Two Tudor-domain containing piRNA factors, Krimper (Krimp) and Qin/Kumo, play crucial roles in making Aub-AGO3 heterotypic ping-pong robust. This maintains the levels of piRNAs loaded onto Piwi and Aub to efficiently repress transposons at transcriptional and post-transcriptional levels, respectively [15].

**Figure 1:**
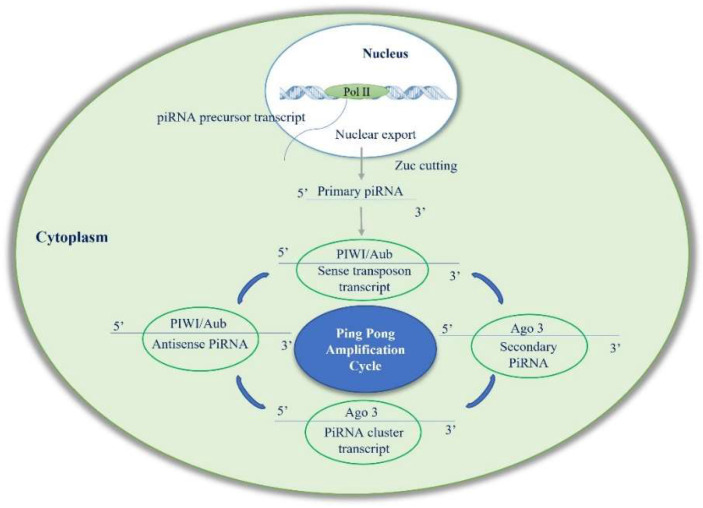
Simplified representation of the currently proposed model of piRNA biogenesis. Two steps of piRNA biogenesis: (1) primary amplification cycle in nucleus and (2) secondary amplification cycle described as “ping-pong cycle” in cytoplasm. Abbreviations: Pol II: polymerase II; Zuc: endonuclease Zucchini; Aub: Aubergine protein; Ago 3: Argonauta 3 protein.

**Figure 2:**
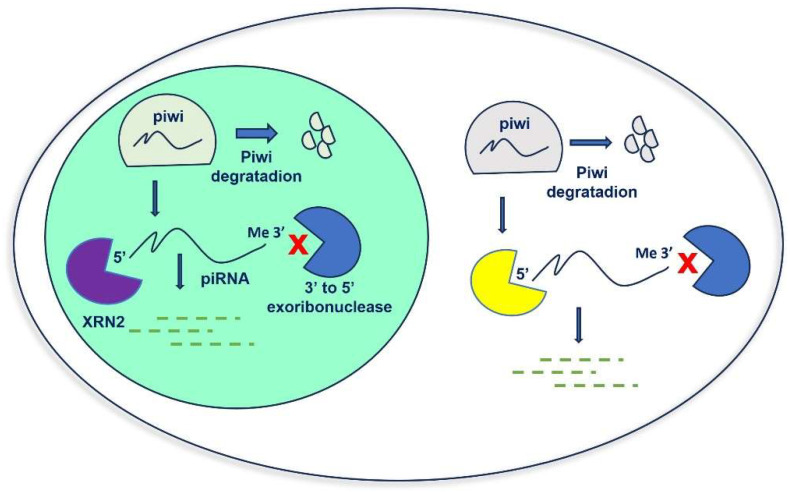
Homeostasis of piRNA in human cells. The degradation of human piRNAs depends on 5′-3′ exoribonuclease.

To summarize, in the primary pathway, piRNA forms a complex with PIWI proteins to mediate transposon silencing. In the secondary pathway, piRNA binds to Aub protein to produce secondary piRNA, while secondary piRNA binds to Ago3 to produce primary piRNA, and the cycle continues [9] (Figure 1). Although with some differences, the piRNA ping-pong mechanism exists not only in germ cells but also in somatic cells [14]. Despite increasing interest in the role of piRNAs in human diseases, their homeostasis in cells is still a poorly understood process. RNAs’ cellular concentration is maintained by a balance of biogenesis and degradation. PIWI proteins protect piRNAs from this degradation. However, when piRNAs are released from the PIWI complex, their 5′ end and 3′ end become unprotected and can easily be accessed by exoribonucleases. The degradation of human piRNAs is mainly dependent on the 5′-3′ exoribonuclease pathway mediated by XRN1 and XRN2, the two major 5′-3′ exoribonucleases involved in piRNA degradation in human somatic cells (Figure 2). It was also reported that the presence of 3′-end 2′-O-methylation in piRNAs reduced their degradation through an exosome-mediated decay pathway [16].

## 3. Function of piRNAs and PIWI Proteins

**Figure 3:**
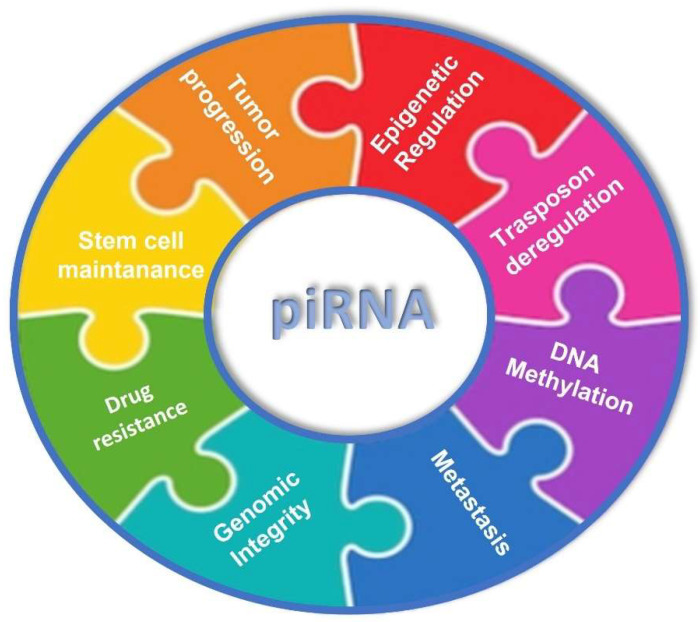
piRNA involvement in different biological processes, hallmarks of cancer, such as tumor progression, epigenetic regulation, transposon deregulation, DNA methylation, metastasis, genomic integrity, drug resistance, and stem cell maintenance.

Depending on the context, piRNAs can act in the following different ways: transposon silencing, epigenetic regulation, germ stem cell maintenance, and genome rearrangement. Recently, piRNAs have been described in tumorigenesis and in various steps of cancer progression, such as proliferation, invasion, metastasis, apoptosis, and drug resistance [17] (Figure 3). piRNAs act in reproduction, in a PIWI-dependent manner, and in fertility regulation by attaching to PIWI proteins and forming a silencing ribonucleoprotein complex [9]. It seems likely their ancestral function was an adaptive mechanism to silence active transposable elements (TEs). Through complementary sequences, piRNA cluster transcripts recognize TEs, avoiding their expression, which might lead to a loss of genome integrity. piRNAs can regulate cellular processes with different mechanisms, also independently of PIWI proteins, such as increasing translation or stabilizing mRNA [18]. Recent findings on piRNAs’ biological significance suggest that they can somatically regulate gene expression also via epigenetic alterations [19]. Epigenetic global changes in cancer include DNA methylation, DNA hypomethylation, CpG island methylation, and gene-specific DNA hypermethylation, leading to oncogene activation (Ras, cyclin D2) [20] and tumor suppressor silencing (RB1, p16) [21]. It was demonstrated that aberrant DNA methylation in tumor cells is linked to PIWI/piRNA disorders. Therefore, piRNA deregulation may influence the expression and stability of the genome, causing cell signaling alteration, which, in turn, may induce disease onset and progression [22,23,24,25,26,27,28].

**Figure 4:**
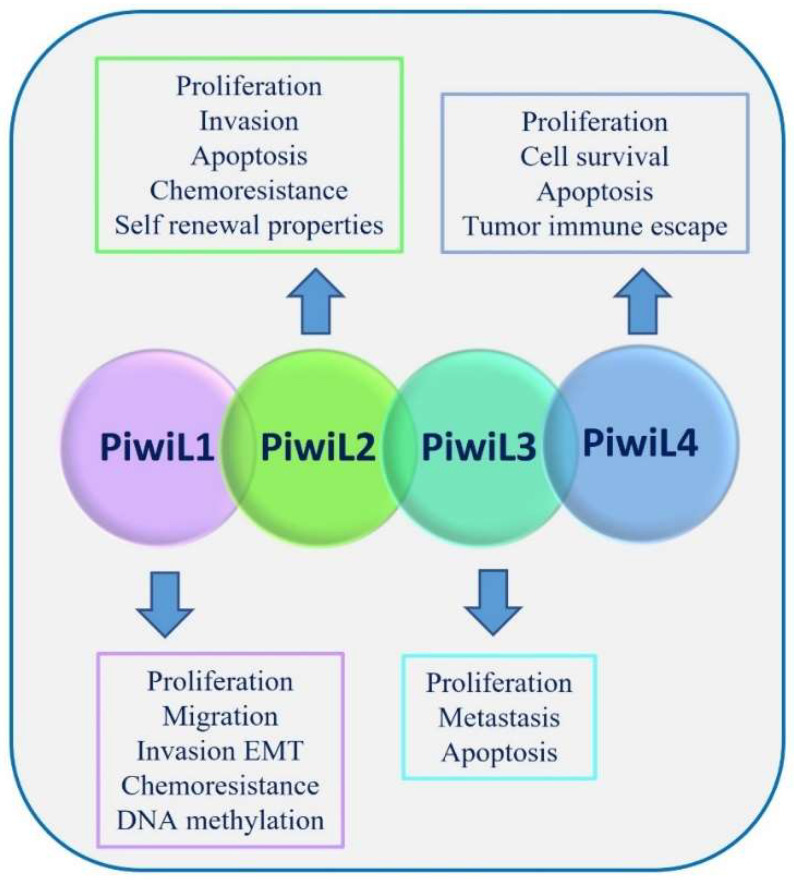
Roles of the four members of PIWI protein family. PIWI1, PIWI2, PIWI3, and PIWI4 have several roles in the steps of cancer progression (such as proliferation, migration, invasion, cell survival, tumor escape, chemoresistance, self-renewal properties, metastasis, apoptosis, EMT: epithelial–mesenchymal transition) and epigenetic regulation (DNA methylation).

PIWI family proteins consist of four members crucial for the biogenesis and function of small ncRNAs: PIWIL1, PIWIL2, PIWIL3, and PIWIL4. PIWIL proteins bind piRNAs, as a unique type of small ncRNA, forming a PIWI/piRNA complex. This complex exerts the gene regulation function, playing an important role in the stability and integrity maintenance of the germ cell genome [8]. PIWI/piRNA acts as an epigenetic modulator recruiting other epigenetic regulatory factors, such as DNA methylase, beyond its function of directly cutting and degrading target RNA, acting like an Ago protein/miRNA complex. PIWI family proteins have been considered prognostic markers for various malignancies [29]. Although specific mechanisms need further investigation, various studies have demonstrated that PIWI proteins, expressed in many cancers, affect multiple biological processes, also without interacting with piRNAs, including different steps of cancer progression such as cell proliferation, apoptosis, migration, invasion, cell cycle regulation, and self-renewal [30] (Figure 4). Specific examples of PIWIL proteins involved in cancer are described below. PIWIL1 is the most studied PIWI protein that regulates gene expression, apoptosis, cell cycle, and proliferation. PIWIL1 is a coactivator of adenomatous polyposis coli C-terminal domain C complex that targets cell adhesion protein, Pinin, for proteolytic ubiquitination, thus promoting metastasis in pancreatic cancer (PC) [31]. PIWIL2 overexpression functions as an oncogene; its deregulation plays an important role in cancer progression and is associated with poor survival and aggressive clinicopathological properties of patients [32]. The deregulation of PIWIL3 is reported in many cancer types; it is highly expressed in both primary ovarian cancer (OC) and metastatic tissues [33]. PIWIL3 plays an important role in melanoma, and its expression correlates with the tumor stage [34]. In gastric cancer (GC), PIWIL3 upregulation increases cell proliferation, migration, and invasion [35]. Conversely, PIWIL3 overexpression seems to have a protective effect in glioma cell lines and decreased tumor size in vivo [36]. Moreover, PIWIL3 is considered a prognostic biomarker of breast cancer (BC) since its upregulation is significantly associated with poor overall survival [37].

PIWIL4 is involved in chromatin modifications in human somatic cells [38], and it can process precursor hairpins generating miRNAs in DICER independent manner [39]. PIWIL4 role in tumorigenesis is controversial; high expressions of PIWIL4 are found in colorectal, cervical, gastric, and ovarian cancer [40,41]. However, other studies reported that low PIWIL4 expression is associated with a poor prognosis in different cancer types [37]. Furthermore, the lack of PIWIL4 expression triggered by CpG island hypermethylation has been found in testicular tumors [42] (Table 1).

## 4. piRNAs in Cancer

Cancer and germ cells share important biological characteristics such as rapid proliferation and the ability for self-renewal. Recently, a growing number of studies have revealed the role of piRNAs in cancers, launching a new biological concept in which piRNAs mediate a gene regulation mechanism typical of germline cells in somatic cells [43]. It is plausible that cancer cells with a high proliferation rate can adopt and utilize self-renewal machinery like germ cells. In malignant cells, piRNAs are involved in epigenetic regulation and may be crucial to sustaining cancer stemness [30].

### 4.1. Role of piRNAs in Cancer Initiation and Progression

**Figure 5:**
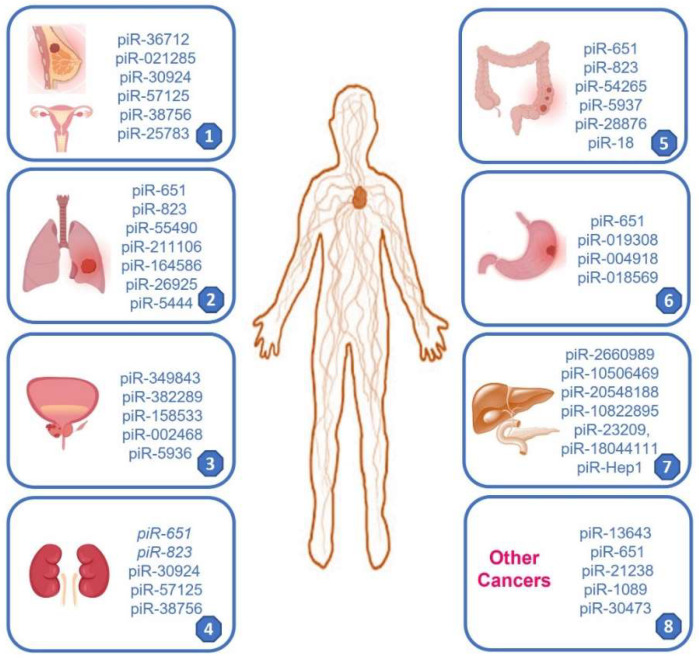
Schematic representation of the piRNAs localization in various cancer types. 1: Breast and ovarian cancer; 2: lung cancer; 3: prostate cancer; 4: renal cancer; 5: colon rectal cancer; 6: gastric cancer; 7: pancreatic and biliary cancer; 8: other cancer type.

Several piRNAs affect cancer stem cells (CSCs) and somatic cells by regulating gene expression via epigenetic processes. CSCs are a small population of cancer cells with high heterogeneity and a great capacity to renew tumors. Aberrant expressions of piRNAs and PIWI proteins also in CSCs can regulate tumor initiation and progression. Examples of specific piRNAs involved in cancer are described below. In non-small cell lung cancer (NSCLC), piR-651 upregulation correlates with a significant increase in tumor growth and metastasis, affecting cell cycle arrest and inducing cyclin D1 and CDK4 and suggesting piR-651 as a potential oncogene. piR-651 overexpression promotes proliferation and invasion and reduces cell apoptosis by inducing different oncogene (CDK4, Cyclin D1, and MDM2) expressions [44]. In addition, piR-651 can increase phosphatase and tensin homolog (PTEN) methylation via DNA (cytosine-5)-methyltransferase 1 (DNMT1) [45]. It was also demonstrated that piR-651 is down-regulated in patients with Hodgkin lymphoma (HL) with respect to healthy controls; low levels of piR-651 correlate with poor prognosis in HL patients [46]. High expressions of piR-30473 support the aggressive phenotype of diffuse large B-cell lymphoma, exerting its oncogenic role through a mechanism involving the upregulation of Wilms Tumor-1 Associated Protein (WTAP), an m6A mRNA methylase, that enhances the global m6A level. WTAP induces the expression of its critical target gene, hexokinase 2 (HK2), by enhancing HK2 m6A level, thereby promoting lymphoma progression [47]. An imbalance in piRNA regulatory processes modifies several levels of gene regulation that control DNA damage repair, chromatin organization, and mitogenic signals, inducing uncontrolled cell proliferation [48]. piR-55490 expression is downregulated in lung cancer (LC), restoring piR-55490 can reduce LC cell proliferation rates, whereas suppressing piR-55490 increases cell proliferation rates. piR-55490 acts by inhibiting the serine/threonine kinase 1 (AKT)/mTOR pathway, thereby suppressing cell growth [49]. piR-211106 binding to pyruvate carboxylase can inhibit the progression of LC-enhancing chemotherapy sensitivity, suggesting that it is a potential therapeutic target [50]. In BC has been described an aberrant expression of various piRNAs. piR-36712 is lowly expressed in this cancer type compared with non-tumor tissues and acts as a possible tumor suppressor. It was also reported that piR-36712 upregulation has a synergistic anticancer effect with chemotherapy on BC cells via the Interaction with SEPW1 pseudogene SEPW1P RNA [51]. piR-36712 can be considered a novel tumor suppressor and a prognostic predictor of BC. Moreover, piR-36712 modulates the expression levels of tumor suppressor genes p53 and P21; its increase leads to cell cycle arrest in the G0/G1 phase of cancer cells [52]. piR-021285 induces methylation at cancer-relevant genes, and it is considered a potential modulator of BC invasiveness by remodeling the cancer epigenome. The exogenous expression of piR-021285 induces significant methylation differences at BC-related genes, including the attenuated methylation of 5′ UTR first exon at the pro-invasive ARHGAP11A gene. There is an increased ARHGAP11A mRNA expression and enhanced invasiveness in variant versus WT piR-021285 mimic-transfected BC cell lines, supporting the role of this piRNA in tumorigenesis via a piRNA-mediated epigenetic mechanism [53]. In clear cells renal carcinoma (ccRC), by using piRNA microarray in a large cohort study, three piRNAs (piR-30924, piR-57125, and piR-38756) have been identified as piRNAs significantly associated with tumor recurrence and overall survival [54]. In cells and plasma of various cancer patients, an altered expression of piR-823 has been observed, with a role in regulating tumor cell growth. In GC, piR-823 acts as a tumor suppressor, and its expression is dramatically decreased in GC tissues. The restoration of piR-823 in GC cells inhibits cancer cell growth both in vitro and in vivo [55]. Also, in colorectal cancer (CRC), piR-823 downregulation inhibits cell proliferation and increases cell apoptosis by inducing an apoptosis activator gene, the transcription factor HSF1 [56]. Moreover, in CRC tissue and serum, piR-54265 is upregulated and induces cancer progression activating STAT3 signaling [57]. Furthermore, it was reported that piR-18 is involved and contributed to the tumorigenesis and progression of CRC. The overexpression of piR-18 inhibits the cell proliferation, migration, and invasion of CRC; thus, it could potentially be used as a new biomarker for diagnosis and therapy [52]. In hepatocellular carcinoma (HCC), a new piRNA, piR-Hep1 has been identified; it is upregulated in HCC with respect to non-tumoral liver cells. The silencing of piR-Hep1 inhibits cell viability, migration, and invasion, with a concomitant decrease in AKT phosphorylation [58,59] (Figure 5).

In BC, piR-2158 is downregulated in CSCs; it was demonstrated that the overexpression of piR-2158 prevents mammary gland tumorigenesis via regulating CSCs and tumor angiogenesis. piR-2158 acts as a transcriptional repressor of Interleukin 11 (IL11) by competing with AP-1 transcription factor subunit FOSL1 to bind the promoter of IL11. piR-2158 can also provide a potential therapeutic strategy in BC treatment [60]. Moreover, the piRNA/PIWI complex can selectively control the phosphorylation of target proteins. It was reported that piR-54265-binding PIWIL2 promotes the formation of PIWIL2/STAT3/phosphorylated SRC complex, inducing phosphorylated SRC-mediated STAT3 phosphorylation that, in turn, causes the proliferation, metastasis, and chemotherapy resistance of CRC cells [19]. The piR-823/PIWIL2 complex mediates STAT3 phosphorylation and the activation of the STAT3/BCL-xl/cyclin D1 pathway, inducing the expression of cyclin-dependent kinase inhibitors and controlling G1 phase regulators Cyclin D1 and CDK4, thus promoting CRC progression [39]. piRNA/PIWI complex can interact with other ncRNAs, including miRNAs and lncRNAs, to regulate cancer progression. piR-30188/PIWIL3 binds to OIP5-AS1, a cancer-associated lncRNA, a target of miR-367-3p in gliomas. miR-367-3p negatively regulates CEBPA mRNA expression and increases TRAF4 expression [36]. The combination of OIP5-AS1 knockdown with the over-expression of PIWIL3 and miR-367-3p leads to tumor regression, identifying a novel molecular pathway in glioma cells that may provide a potential innovative approach for cancer therapy [23]. Overall, these findings indicate that piRNAs can have several potential clinical applications in diagnosis, prognosis, and therapy (Table 2).

### 4.2. piRNAs as Cancer Biomarkers

ncRNAs have received a lot of attention as one factor contributing to genetic and epigenetic instability. Clinically, ncRNA alterations have a relevant diagnostic and prognostic significance [26]. Epigenetic biomarkers can be useful in predicting therapeutic drug responses, and piRNAs are considered emerging biomarkers for therapy monitoring. A biomarker is a significant indicator that can be used to assess a target’s diagnostic potential, risk of recurrence, and clinical prognosis [63]. Aberrant expressions of piRNAs and their correlation with cancer patient features suggest that these may have an important clinical impact, not only as diagnostic biomarkers but also as druggable targets [67]. Alterated piRNA levels may be considered good cancer biomarkers, with higher sensitivity and specificity than miRNAs [69]. Nowadays, few studies investigate the role of piRNAs as cancer biomarkers, but the field is in progress and updated [70]. Some studies indicate that piRNAs have a better diagnostic ability than traditional biomarkers. It was reported that piR-13643 and piR-21238 performed better than conventional biomarkers, such as hector battifora mesothelial antigen-1 (HBME1), conventionally used to discriminate malignant nodules from benign ones in papillary thyroid carcinoma [65]. Moreover, serum piR-5937 and piR-28876 were able to distinguish CRC from healthy controls with higher sensitivity and specificity than traditional markers such as Carcinoembryonic Antigen (CEA) and Carbohydrate antigen 19-9 (CA199) [61]. It was also suggested that piR-54265 can be used as a biomarker for the early detection and clinical monitoring of CRC [77].

## 5. piRNAs and Liquid Biopsy

Liquid biopsy is considered an ideal tool for discovering new cancer biomarkers, and in the current era of personalized medicine, LB has acquired high relevance in cancer patient management [66]. LB serves as a safe alternative to solid biopsies; its main components are circulating tumor cells (CTCs), circulating tumor DNA (ctDNA), circulating tumor RNA (ctRNA), tumor-associated platelets, and EVs [78]. Nowadays, clinical oncology adopts next-generation sequencing (NGS)-based diagnostics; these high throughput technologies allow for the identification of ncRNA profiles and significant genetic mutations across the human genome. LB is increasingly being used for early diagnosis and to determine the best therapeutic option for cancer patients [79].

### 5.1. piRNAs in Extracellular Vesicles

Extracellular vesicles (EVs) are nanoscale membrane particles released by all cytotypes in physiological and pathological conditions. EVs are internalized by target cells through a different mechanism and transport a plethora of bioactive molecules, including proteins, lipids, and nucleic acids [68,75,80,81,82]. EVs have a key role in several steps of cancer progression; they are involved in proliferation, migration, premetastatic niche formation, and immune response [62,76]. EVs have been reported to contain an abundance of RNAs, such as ncRNAs, including miRNAs, lncRNAs, circRNAs, and piRNAs [74]. The packaging of different ncRNAs into EVs is selective and mirrors the status of parental cells. EV-RNAs, with regulatory effects, have been implicated in many EV-mediated biological functions [64,75]. EVs are widely present in biofluids, such as blood, urine, saliva, and malignant effusions. Moreover, piRNAs can cross the plasma membrane, and EVs released by cancer cells contained piRNAs that remain stable in body fluids. This feature suggests that piRNAs can easily be detected in body fluids as accurate biomarkers [44]. Several studies have focused on plasma EV’s role as an early and non-invasive diagnostic biomarker for cancers. EV-cargo represents a valuable source of genetic materials, mainly ncRNA biomarkers [83]. These findings indicate that LB can allow for the detection of piRNAs that may become therapeutic and diagnostic tools for various cancer types. Differentially expressed EV-piRNAs have been identified in patients with specific disease conditions compared to healthy controls, suggesting an association between piRNA and progression in various diseases [72]. Although we are currently lacking guidelines on piRNA bioinformatic analysis, EV-piRNA can be considered a new research niche in human cancer pathology. piRNAs encapsulated in EVs are stable in body fluids and have the potential to be promising markers for cancer diagnosis and prognosis and new tools for innovative therapies [73]. Examples of specific piRNAs shuttled by EVs are described below and summarized in Table 3.

It was reported that piR-26925 and piR-5444 are upregulated In EVs isolated from the serum of LC patients [84]. A high-throughput sequencing of small ncRNAs from EVs, cancerous and adjacent noncancerous tissues in patients with NSCLC, was applied to recognize candidate piRNAs as diagnostic biomarkers. This study reveals that piR-164586 was significantly upregulated in paracancerous tissues and EVs from serum samples of healthy individuals [85].

Recently, it was demonstrated that EVs isolated from the plasma of neuroblastoma (NB) patients contain high levels of piR-1089. In vitro, studies indicated that EV-piR-1089 promoted NB cell proliferation and migration by inhibiting Kelch-like ECH-associated protein 1 (*KEAP1*) expression. Low *KEAP1* expressions were associated with NB progression in clinical samples [86]. piRNAs are also involved in the crosstalk between multiple myeloma (MM) cells and bone marrow microenvironment. In MM, piR-823 silencing causes cell cycle dysregulation, reduction of apoptosis-related proteins, and de novo DNA methyltransferases, thus inhibiting the tumorigenicity of MM in vitro and in vivo [87]. piR-823 contained in EVs from peripheral blood of MM patients and cell lines plays an important role in cell–cell communication between endothelial and myeloma cells in the tumor microenvironment. EV-piR-823 from MM cells can be effectively transferred to endothelial cells and alter their biological characteristics, promoting anti-apoptotic and pro-angiogenic activity and creating a favorable microenvironment for MM cell survival. The upregulation of piR-823 can increase the expressions of IL-6, VEGF, and ICAM-1, promoting endothelial cell proliferation, invasion, and tube formation [88]. Granulocytic myeloid-derived suppressor cells (MDSCs) can trigger piR-823 expression, and silencing piR-823 can decrease the stemness of MM stem cells maintained by granulocytic MDSCs [89]. Moreover, piR-823 expression has been correlated with the stage and prognosis of MM, indicating its potential as a therapeutic target and prognostic stratification biomarker [88,90]. Gu et al. reported that the EV-piRNA population is altered in the plasma of cholangiocarcinoma (CCA) and gallbladder carcinoma (GBC) patients compared to healthy individuals. EV-piRNA profiling revealed unique piRNA signatures of CCA and GBC. Small RNA sequencing data, obtained by the NGS system, showed piR-2660989, piR-10506469, piR-20548188, piR-10822895, piR-23209, and piR-18044111 in EVs isolated from CCA and GBC plasma. Interestingly, expression level analyses of EV-piRNAs in plasma of CCA and GBC patients, before and after surgeries, have demonstrated that piR-10506469 and piR-20548188 were significantly decreased in patients who underwent surgeries [91]. In OC, EVs drive the crosstalk between cancer cells and omental fibroblasts. EV-piR-25783 activates TGF-β/SMAD2/SMAD3 pathway in fibroblasts and promotes the fibroblast-to-myofibroblast transition in the omentum, secretion of various cytokines, proliferation, migration, and invasion, contributing to the premetastatic niche formation. piR-25783-induced myofibroblasts improve tumor implantation and growth in the omentum. EV-piR-25783 upregulation is thus associated with adverse clinicopathological characteristics and shorter survival [92]. Moreover, EV-piRNAs can be considered promising non-invasive diagnostic biomarkers for GC, piR-019308, piR-004918, and piR-018569 contained in serum EVs were significantly increased in GC patients compared to healthy controls [93].

In prostate cancer (PCa), piRNAs were involved in the proliferation, migration, and invasion of PCa cells by activating different signal pathways, which may represent a new marker of PCa diagnosis. It was reported that piRNAs contained in urinary EVs, isolated from patients with PCa and detected using NGS technology, are useful for diagnosis. The fraction of PCa-derived EVs in urine is larger than in plasma and allows for a better detection and tracking of PCa-derived RNAs [94]. The expressions of piR-349843, piR-382289, piR-158533, and piR-002468 in urinary EVs were significantly increased in PCa patients compared with healthy controls [95]. Interestingly, it was also indicated that in plasma EVs of patients with bladder cancer (BCa), two small ncRNAs, miR-4508 and piR-5936, were associated with the risk class: miR-4508 with a descendant trend going from controls to high-risk BCa; piR-5936 with an ascending trend [96]. Taken together, these reports indicate that EV-piRNAs have an important role in tumor–host crosstalk, contributing to cancer progression and showing a great potential as theranostic biomarkers.

### 5.2. piRNA-Based Therapeutic Approaches in Cancer

On-target specificity is a significant difficulty to overcome for an RNA-based therapeutic strategy that involves gene silencing. Moreover, RNAs are unstable molecules, and the development of ways to enhance RNA stability and its uptake are crucial points. Although siRNA molecules are structurally susceptible to RNases and degradation, some advances to overcome these limits are ongoing, employing various chemical and structural modifications that make RNAi delivery more physiologically amenable. Synthetic lipid nanoparticles have been developed and have enabled successful systemic delivery of RNAi. Each of these RNA components can be modified to overcome their susceptibility to degradation [94]. Stability and barriers to delivery were overcome thanks to the success of the mRNA vaccine for COVID-19 in early 2021, opening new routes for targeted RNA therapies [95]. siRNAs are double-stranded ncRNAs with similar biological functions to miRNAs but are synthetically derived (exogenously) via the process of RNA interference (RNAi). siRNAs, like miRNAs, degrade target mRNAs in a sequence-specific manner, leading to gene silencing. RNAi machinery targets and can silence pathologic mRNA sequences [94].

Some typical limitations of siRNAs and miRNAs can be overcome using piRNAs and EVs as their shuttles. Nowadays, the potential role of piRNAs as therapeutic tools has been reported; piRNA treatment is performed via the correction of pathological alteration of piRNA expression, including the reactivation of endogenous piRNA as a pathological inhibitor and functional blocking or reduction in piRNA expression as a pathological driver [30]. Synthetic piRNAs are very intriguing as they could block the synthesis of cancer-related proteins by binding to mRNAs. Different from miRNAs, which need to be processed by enzymes and regulate several mRNAs, piRNAs have the advantage of not requiring enzyme processing and better specificity than other ncRNAs to targets [97]. Since epigenetic regulatory mechanisms are closely related to cancer progression, the epigenetic reprogramming of cancer cells can be a potentially powerful therapeutic approach [43]. Epigenetic drugs (epidrugs) consist of small molecules targeting epigenetic key signaling pathways, promoting transcriptional and post-transcriptional modifications and acting mainly on tumor suppressor and DNA repair gene activation [44]. Since piRNAs regulate protein-coding genes involved in the development of various diseases, they can be considered molecules useful as innovative epidrugs [45]. Although the studies on piRNAs as new therapeutic methods are still in the early stage, understanding the mechanisms and functions of piRNAs could enhance therapeutic alternatives in cancer. Recently, EV-mediated ncRNA delivery systems have attracted tremendous attention in the research of next-generation therapeutics for the treatment of several pathologies, including cancer [98]. EVs, thanks to their small size, low immunogenicity, and high biocompatibility due to their natural origin [99,100], are more attractive than synthetic vehicles. Moreover, EV cargos are protected from degradation in the bloodstream by a lipid bilayer membrane; they have the potential to escape from immune system clearance and cross the physiological barriers [101]. Despite these advantages, natural EVs show some pitfalls. The procedures for EV isolation are time consuming, monitoring methods for cargo dosage are insufficient, and EV stability after storage is not easy to monitor [102]. Currently, the yield of EVs isolated from body fluids or culture media is insufficient to supply clinical demands, and EV-quality control is a challenge that limits their clinical application [103]. To overcome this limit, EVs collected from non-human sources such as plants, bacteria, and milk are considered a potential alternative for cancer therapy. Since EV-mediated communication is involved in all the domains of life and in several cellular physiological and pathological processes, EVs are involved in a universal, evolutionarily conserved mechanism for inter-kingdom and intra-kingdom communication [104]. It was reported that plant EVs can be used as nanoplatforms to transfer ncRNAs in inter-kingdom communication [28,71]. ncRNAs contained in plant EVs are considered a new class of cross-kingdom modulators, capable of mediating animal-plant interactions at the molecular level [105]. Applications of plant EVs in nanomedicine and nutraceutics are based on their intrinsic biological properties, such as anti-cancer, anti-inflammatory, and anti-aging, and on their use as shuttles of therapeutic biomolecules [106]. It was also reported that bacterial EVs isolated from the host gut microbiome can enter the circulatory system to disseminate to distant organs and tissues and be detected in human biofluids. Bacterial EVs in microbiome-based liquid biopsies might be useful for cancer diagnostics and bioengineering strategies for cancer therapy [107]. Moreover, bovine milk-derived EVs have been shown to increase the oral bioavailability of drugs and are optimal vehicles to transport bioactive compounds for nutritional and therapeutic purposes [108]. In cancer therapy, milk EVs can be functionalized with ligands such as folic acid to achieve cancer targeting [109]. In addition, milk-derived EVs have shown several therapeutic effects, such as a selective interaction with macrophages and an increase in intestinal stem cell proliferation as potential adjuvant therapy to standard clinical management of malnourished children [110].

## 6. Conclusions and Outlook

**Figure 6:**
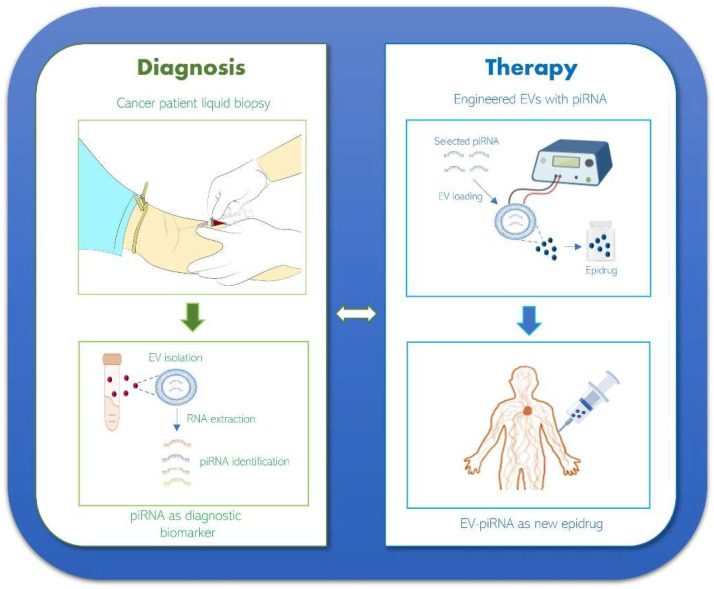
Working hypothesis of piRNA use in diagnosis and therapies. The identification of EV-piRNAs collected by the blood of cancer patients can be useful as diagnostic biomarkers. The engineering of EVs, loading ectopic piRNA, may allow shuttling piRNA useful as new epidrugs.

Recently, significant advances in the piRNA field have been made, providing mechanistic insights into the transcription of piRNA clusters, piRNA biogenesis, and piRNA function. Although the knowledge of piRNAs in cancers remains in its infancy, a growing number of studies have shown that piRNAs can be considered good diagnostic and prognostic markers and new therapeutic tools. However, it needs to report a few studies indicating that piRNAs could be fragments of other ncRNAs and could correspond to false positives due to the use of noncurated piRNA databases [111,112]. Many studies have found aberrant piRNAs in biofluids of cancer patients; thus, LB is considered an ideal tool for developing cancer biomarkers. Even if a single piRNA can be sufficient to distinguish cancer patients from healthy controls, combined biomarkers may be more diagnostically accurate than one. EVs can have a dual role in cancer management as a source of diagnostic biomarkers with the identification of specific piRNAs and as a therapeutic tool consisting of engineered EVs with piRNAs useful as potential and breakthrough epidrugs (Figure 6). Future research efforts should concentrate on wide studies on piRNA profiles in different cancer types and stages. This effort can allow for the identification of comprehensive diagnostic panels able to compliant the heterogeneous landscape of cancer. Preclinical studies show how smart EVs have the potential to expand into next-generation drugs to address the current demand for precision cancer therapies. However, numerous milestones on piRNAs contained in EVs are yet to be achieved. To date, the clinical trials with piRNAs and EV-piRNAs are still a hard challenge, and only a few clinical studies on these emerging ncRNAs are ongoing (www.clinical.trials.org, accessed on 20 April 2023). Further clinical studies are needed to demonstrate that EV-piRNAs are smart tools useful for theranostic applications in precision oncology.

## Figures and Tables

**Table 1:** PIWI protein expression and function in different cancers.

Piwi Protein	Cancer type	Expression	Function	References
PIWIL1	Gasric	Upregulated	Migration proliferation invasion	[32]
	Mieloma Multiple	Upregulated	Chemoresistance	[30]
	Lung	Upregulated	proliferation migration	[31]
	Breast	Upregulated	DNA methylation	[33]
PIWIL2	Colon Rectal	Uperegulation	Chemoresistance Proliferation	[19,22]
	Gastric	Downregulated	Self renewal properties	[19]
	Ovarian	Upregulated	Apoptosis	[20]
	Breast	Upregulated	Chemoresistance	[19]
PIWIL3	Breast	Upregulated	Proliferation Cell survival	[24]
	Ovarian	Metastasis	Metastasis	[20]
	Mieloma Multiple	Upregulated	Apoptosis	[21]
	Gastric	Upregulated	proliferation migration invasion	[22]
	Glioma	Downregulated	Tumor immune escape	[23]
PIWIL4	Lung	Upregulated	Proliferation	[39]
	Liver	Upregulated	Metastasis	[27]
	Gastric	Upregulated	Apoptosis	[28]
	Testicular	Downpregulated	Proliferation	[29]
	Ovarian	Upregulated	Metastasis	[20]

**Table 2:** piRNA localization and their potential clinical applications in different cancers.

Cancer Type	piRNA	Expression	Sample Type	Target	Potential Clinical Application	References
Lung	piR-651	Upregulation	Cell	CDK4 Cyclin D1 MDM2 PTEN DNMT1	Diagnosis	[34,51]
	piR-211106	Upregulation	Cell	Pyruvate carboxylase	Therapy	[56]
	piR-55490	Downregulation	Cell	AKT mTOR	Therapy	[55]
	piR-5444	Upregulation	EV		Diagnosis Prognosis Therapy	[61]
	piR-26925	Upregulation	EV		Diagnosis Prognosis Therapy	[61]
	piR-164586	Upregulation	EV		Diagnosis	[62]
Gastric	piR-823	Downregulation	Cell Plasma		Diagnosis	[63]
	piR-019308	Upregulation	EV		Diagnosis	[64]
	piR-004918	Upregulation	EV		Diagnosis	[64]
	piR-018569	Upregulation	EV		Diagnosis	[64]
Breast	piR-2158	Downregulation	Stem cell	IL11	Therapy	[65]
	piR-823	Upregulation	Stem cell	DNMT	Therapy	[66]
	piR-36712	Upregulation	Cell	SEPW1 p53 p21	Prognosis	[57,58]
	piR-021285	Upregulation	Cell	ARHGAP11A	Therapy	[53,61]
Colon Rectal	piR-823	Downregulation	Cell	HSF1		[67]
	piR-54265	Upregulation	Tissue Serum	STAT3	Prognosis	[35]
	piR-5937	Upregulation	Serum		Diagnosis	[68]
	piR-28876	Upregulation	Serum		Diagnosis	[68]
	piR-18	Upregulation	Tissue Cell		Diagnosis Therapy	[58]
Liver	piR-Hep1	Upregulation	Tissue Cell	AKT	Diagnosis	[69,70]
Neuroblastoma	piR-1089	Upregulation	EV	KEAP1	Prognosis	[71]
Lymphoma	piR-651	Downregulation	Serum		Prognosis	[52]
Renal	piR-38756	Upregulation	Tissue		Prognosis	[60]
	piR-57125	Upregulation	Tissue		Prognosis	[60]
	piR-30924	Upregulation	Tissue		Prognosis	[60]
B-cell lymphoma	piR-30473	Upregulation	Serum	WTAP HK2	Prognosis	[53]
Bladder	piR-5936	Upregulation	Plasma EV		Diagnosis	[72,73]
Ovarian	piR-25783	Upregulation	Plasma EV		Diagnosis Prognosis	[74]
Thyroid	piR-13643	Upregulation	Tissue		Diagnosis	[75]
	piR-21238	Upregulation	Tissue		Diagnosis	[75]
Cholangio Gallbladder	piR-2660989	Upregulation	EV		Diagnosis Prognosis	[76]
	piR-10506469	Upregulation	EV		Diagnosis Prognosis	[76]
	piR-20548188	Upregulation	EV		Diagnosis Prognosis	[76]
	piR-10822895	Upregulation	EV		Diagnosis Prognosis	[76]
	piR-23209	Upregulation	EV		Diagnosis Prognosis	[76]
	piR-18044111	Upregulation	EV		Diagnosis Prognosis	[76]

**Table 3:** piRNAs content in EVs collected from different cancer types.

Cancer Type	piRNA	EV Origin	Target/Role	References
Lung	piR-26925 piR-5444 piR-164586	Serum	Biomarker	[61,62]
Ovarian	piR-25783	CM	TGF-β/SMAD2 /SMAD3 pathway	[74]
Gastric	piR-019308 piR-004918 piR-018569	Serum	Biomarker	[64]
Prostate	piR-349843 piR-382289 piR-158533 piR-002468	Urine	Biomarker	[72]
Neuroblastoma	piR-1089	Plasma	KEAP1	[71]
Multiple Mieloma	piR-823	CM, Plasma	IL-6, VEGF ICAM-1	[105]
Cholangio Gallbladder carcinoma	piR-2660989 piR-10506469 piR-20548188 piR-10822895 piR23209 piR-18044111	Plasma	Biomarker	[76]
Bladder	piR-5936	Plasma	Biomarker	[73]

## Data Availability

Not applicable.
